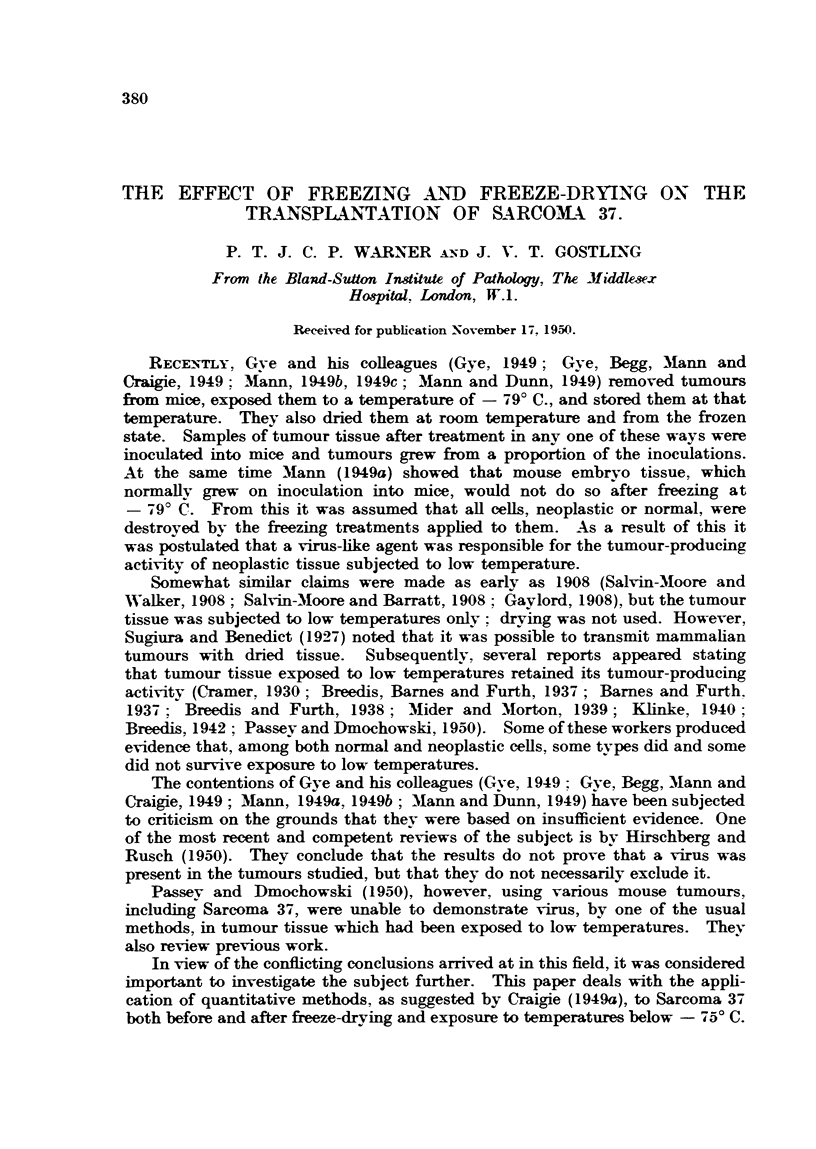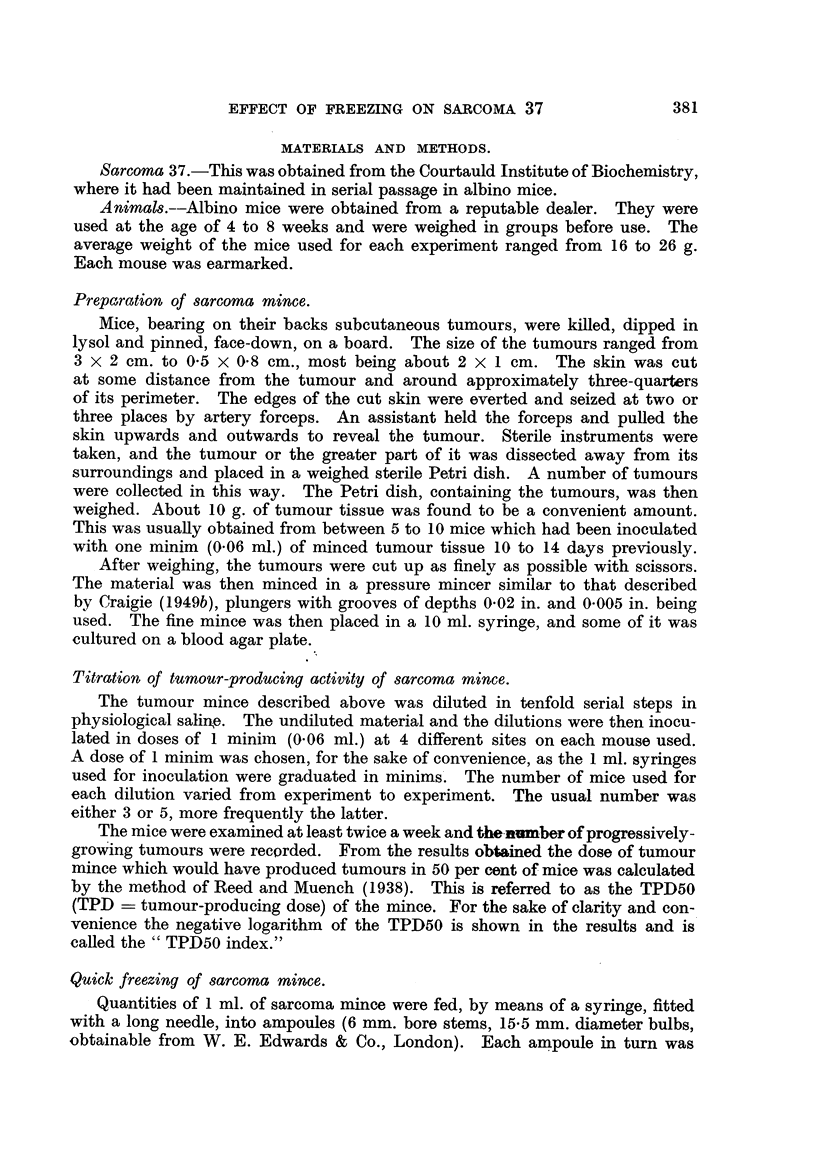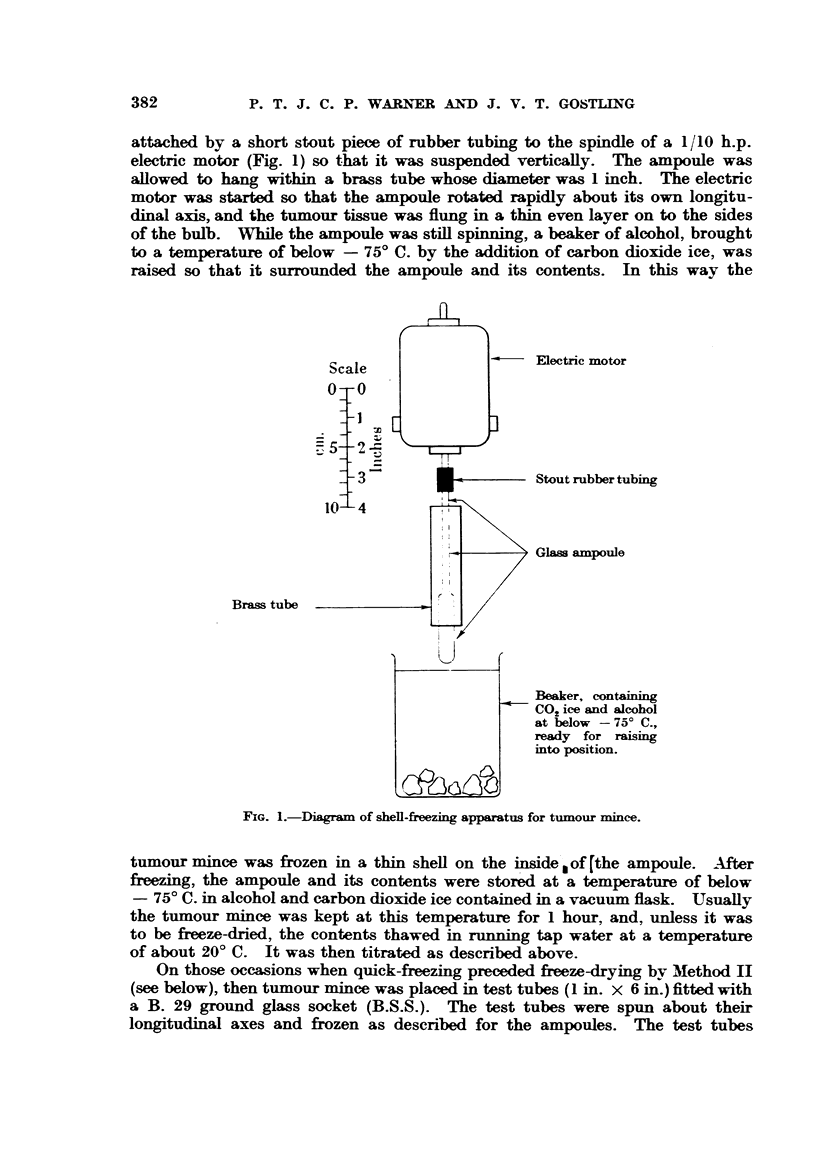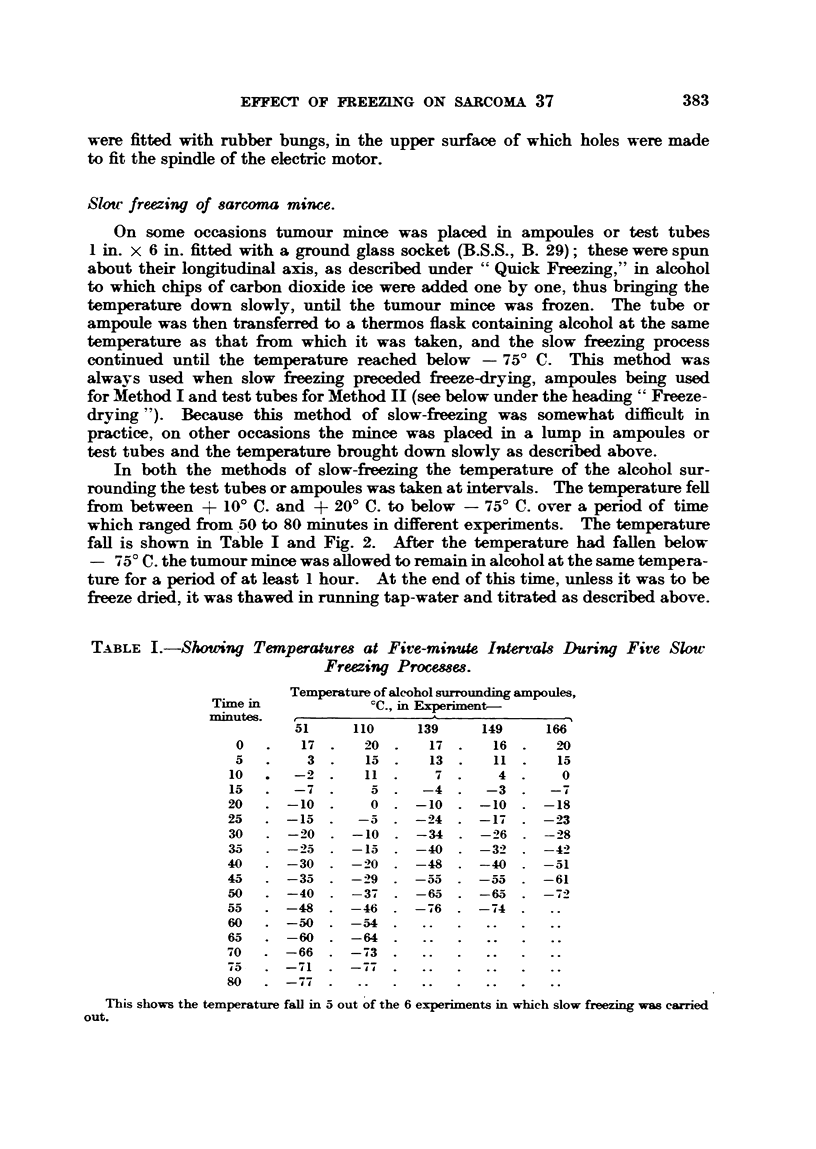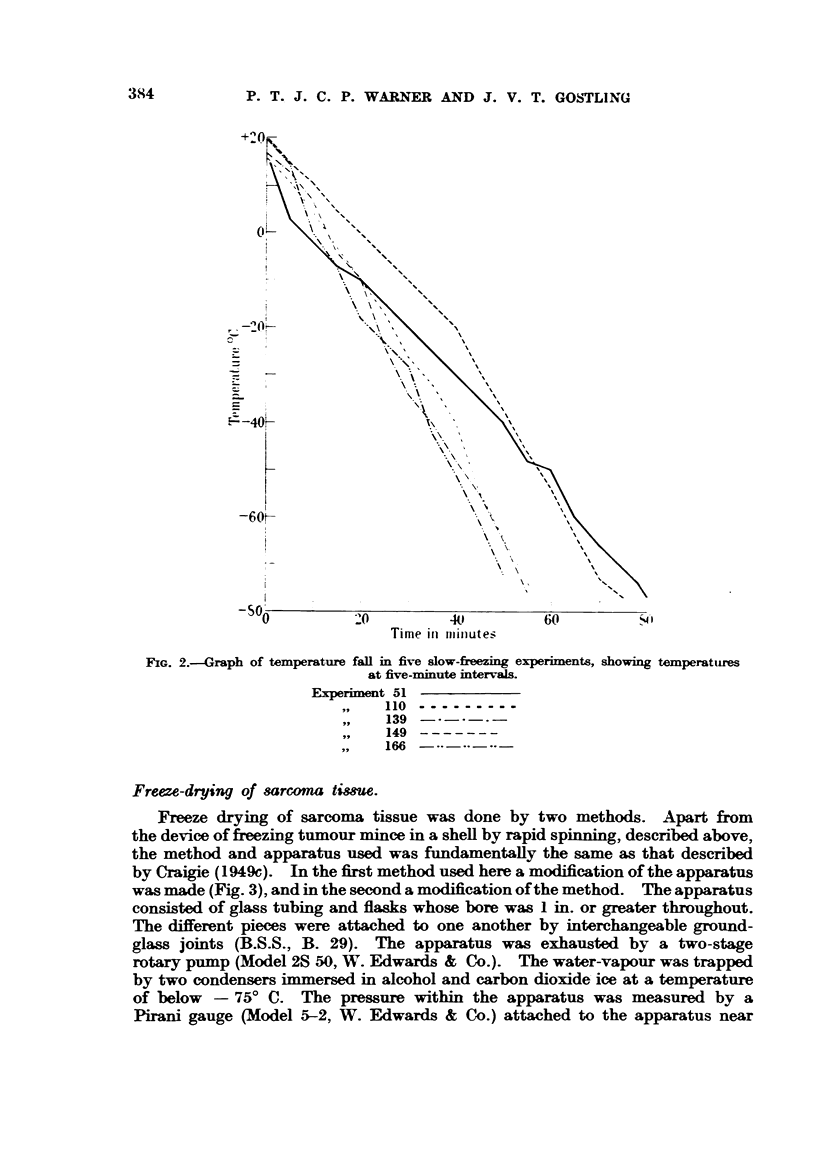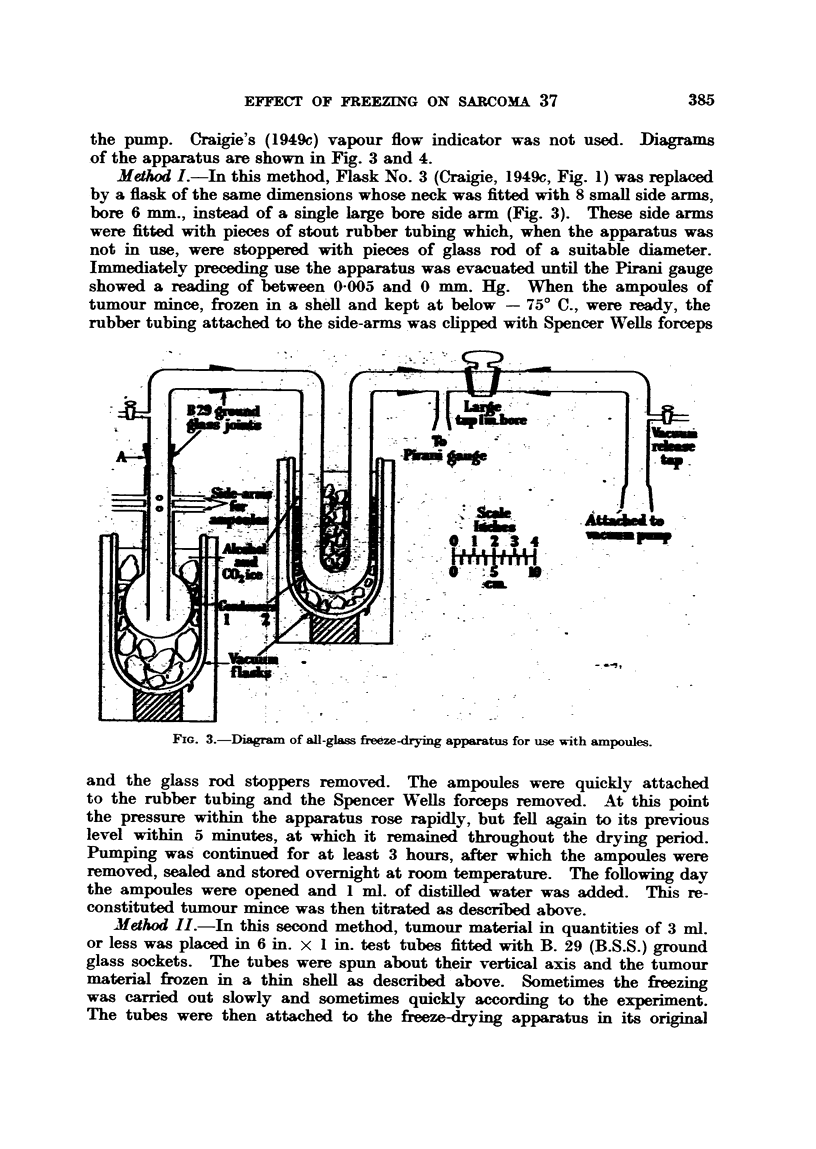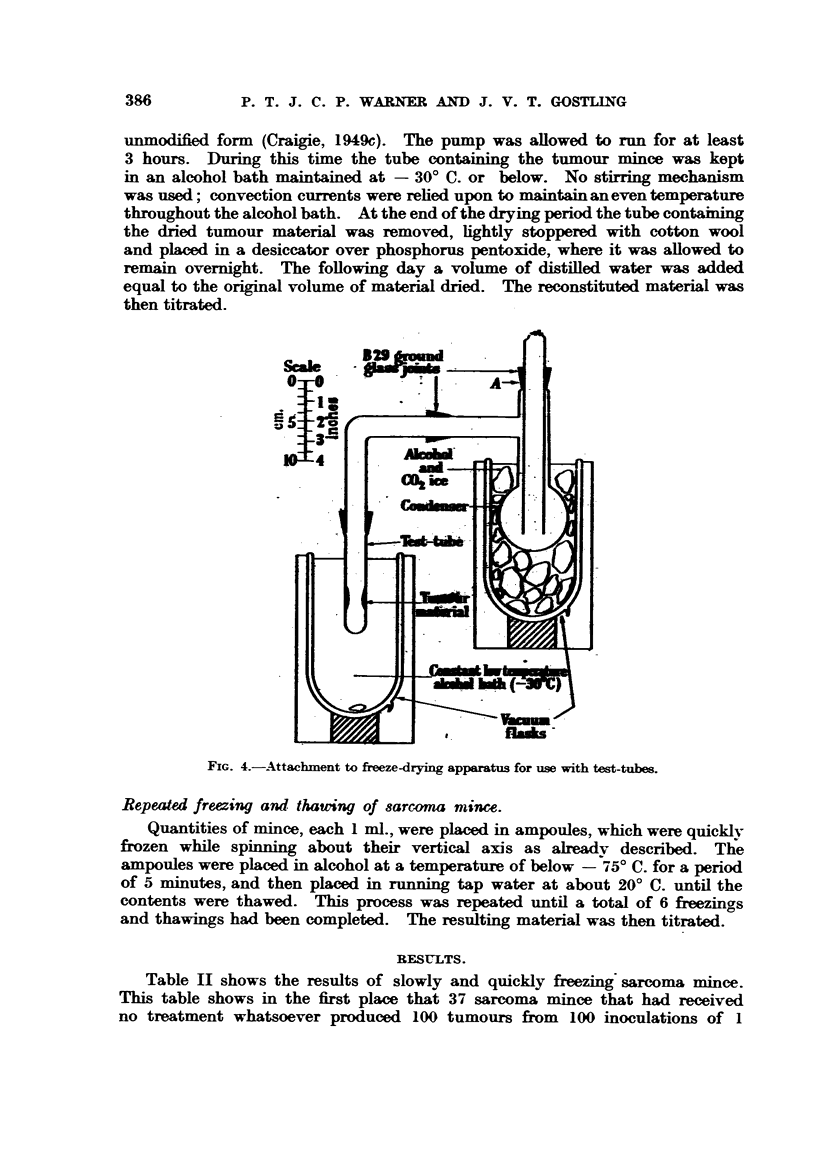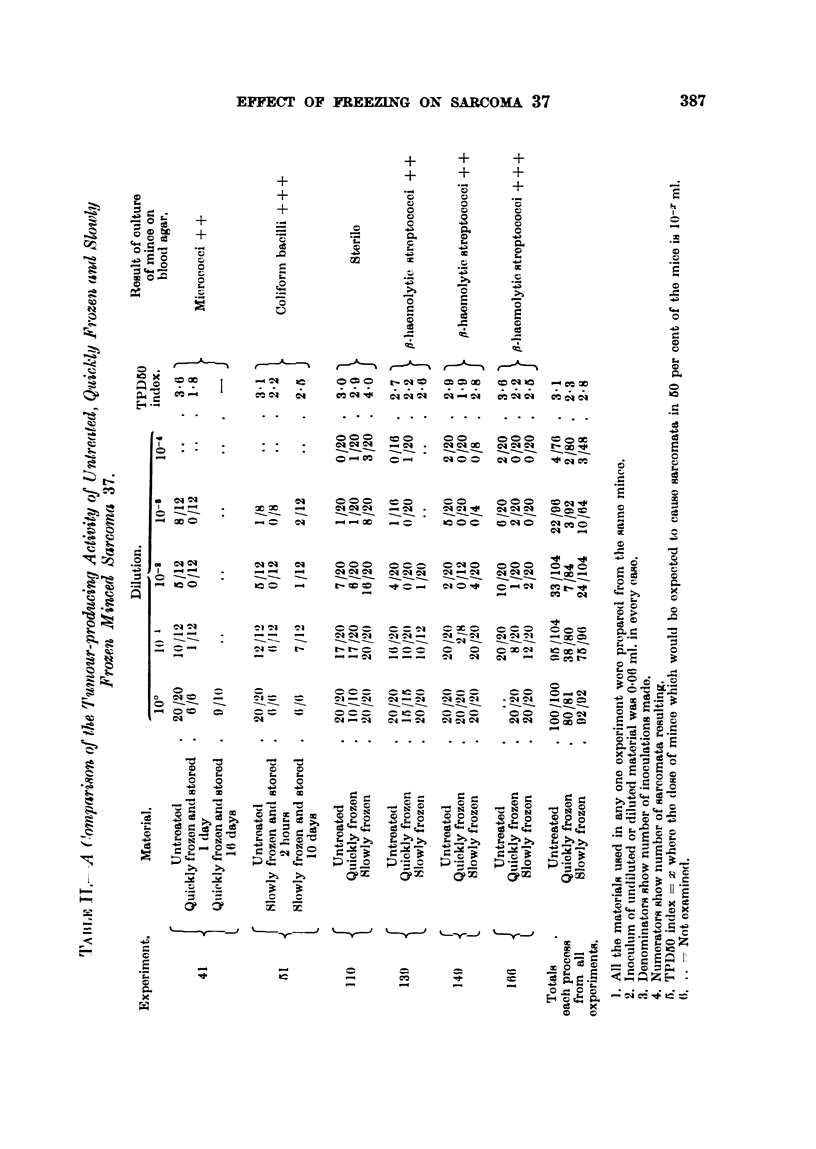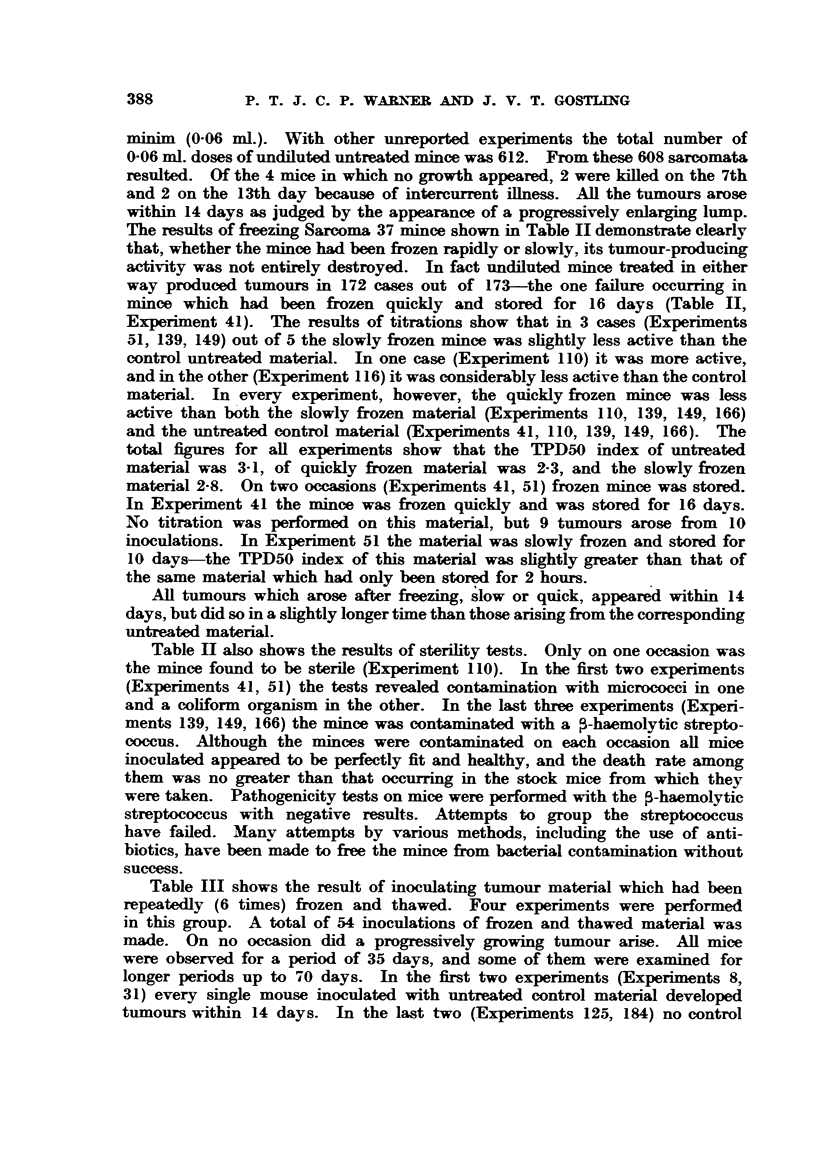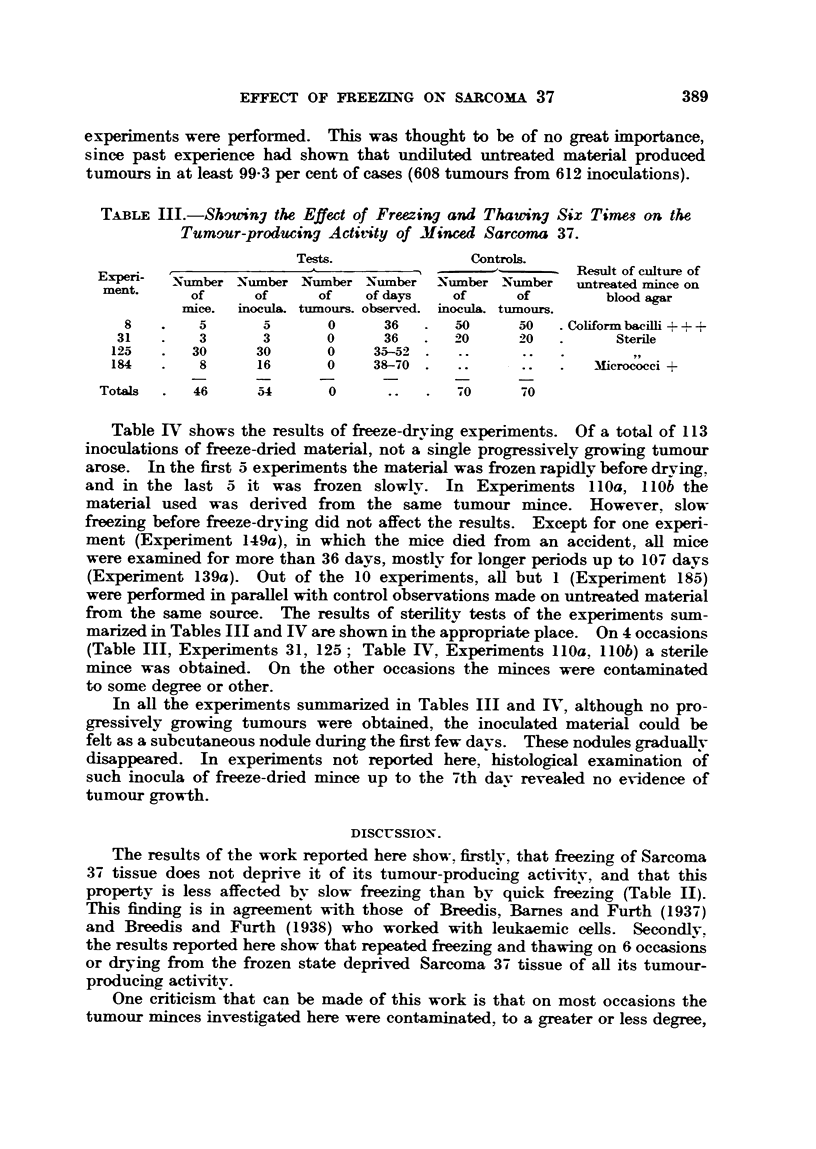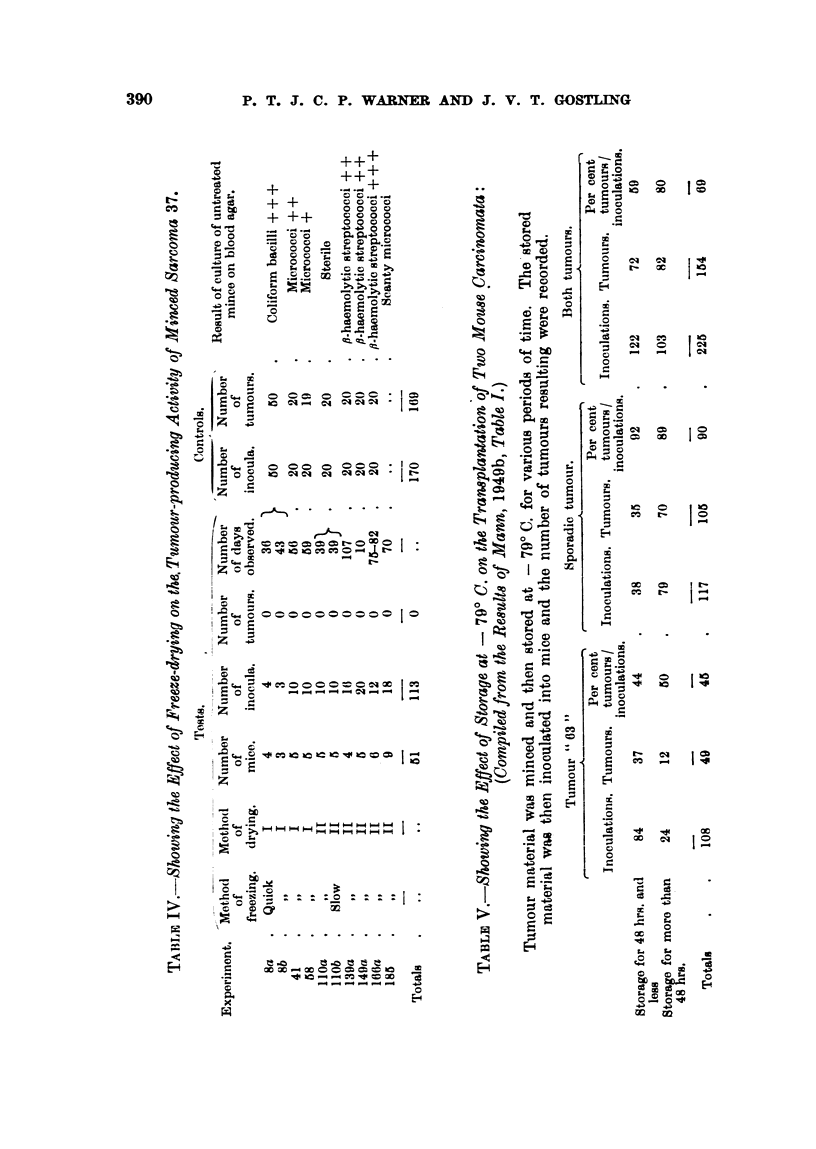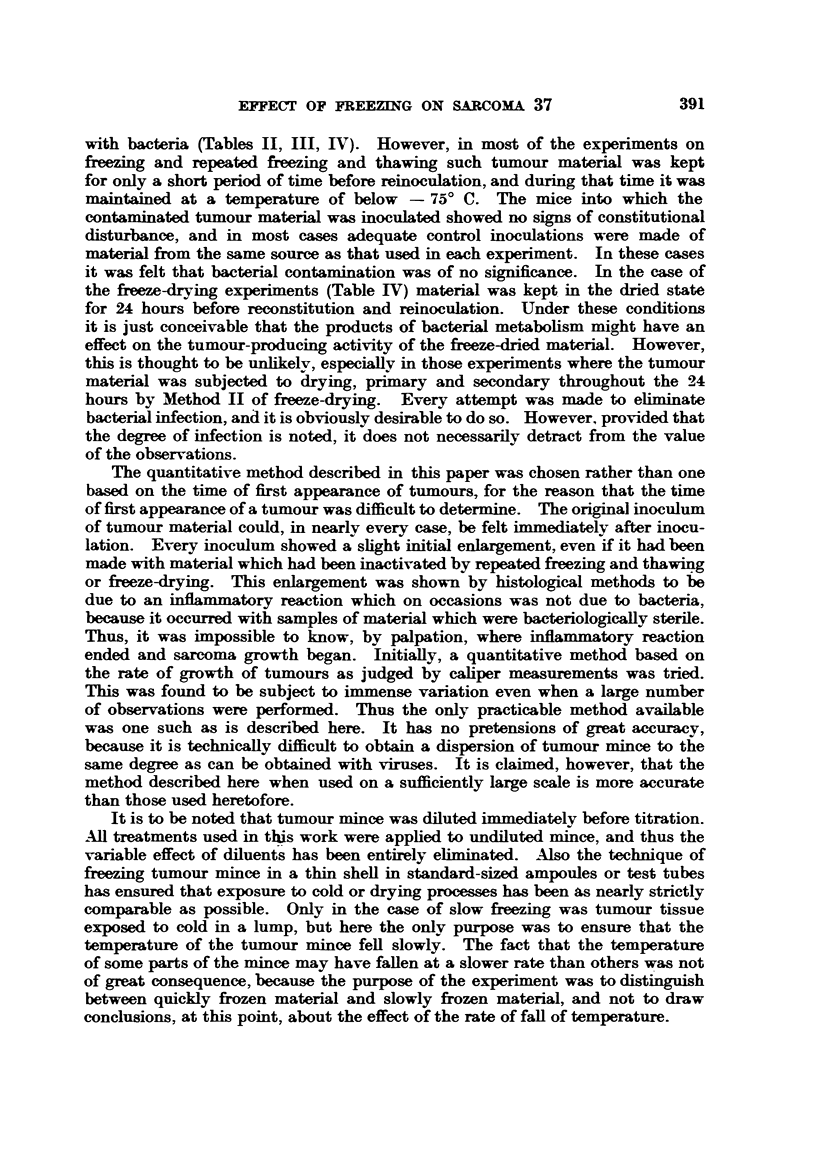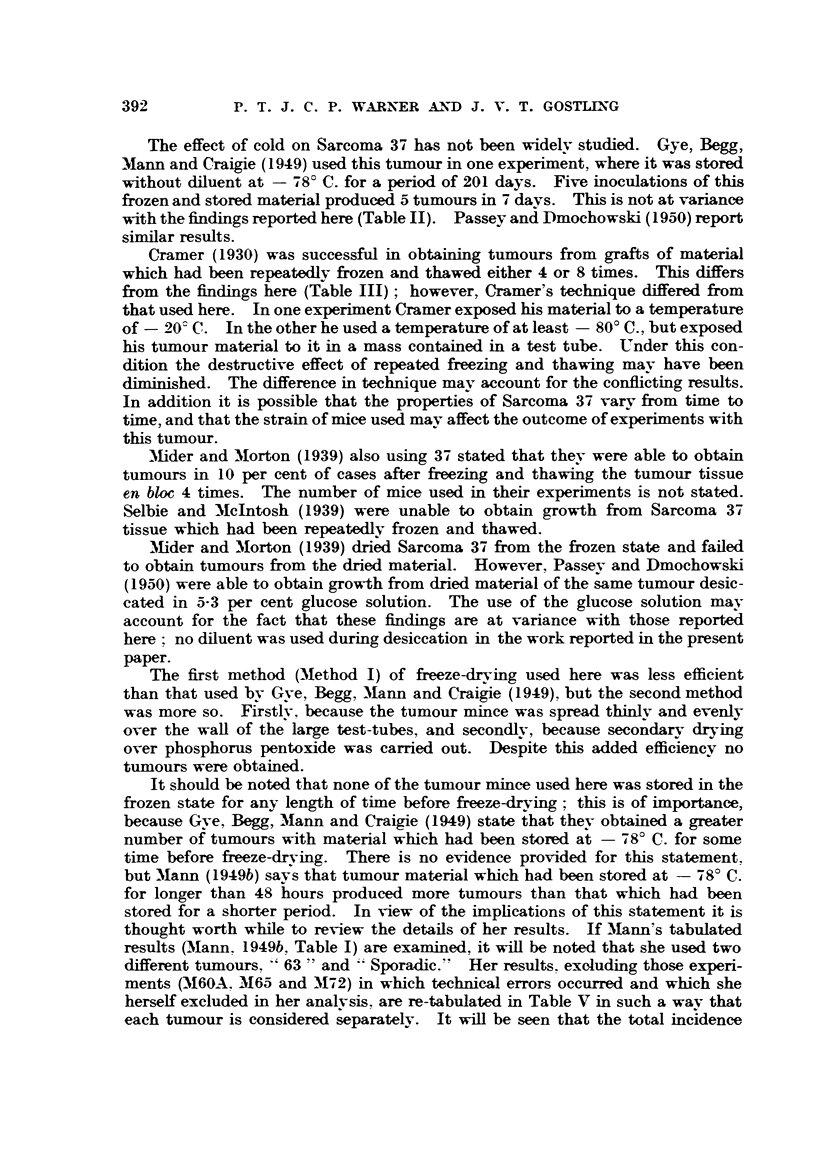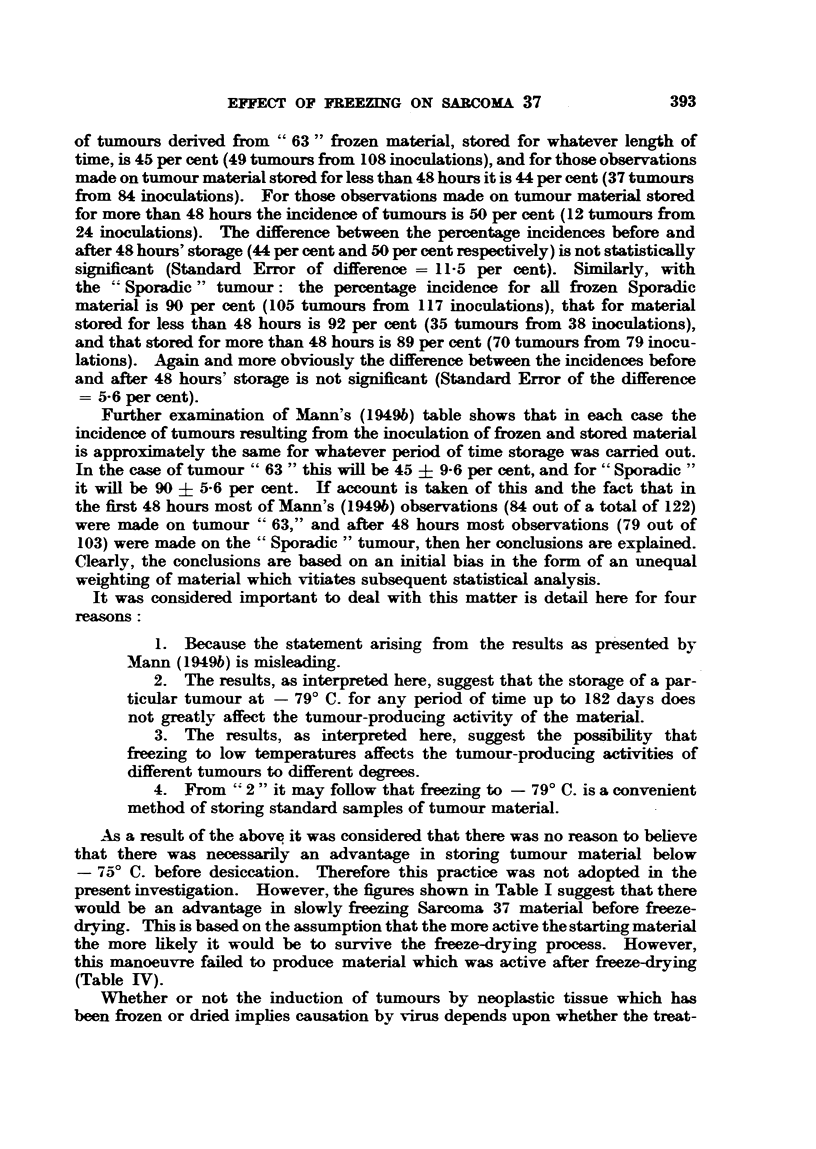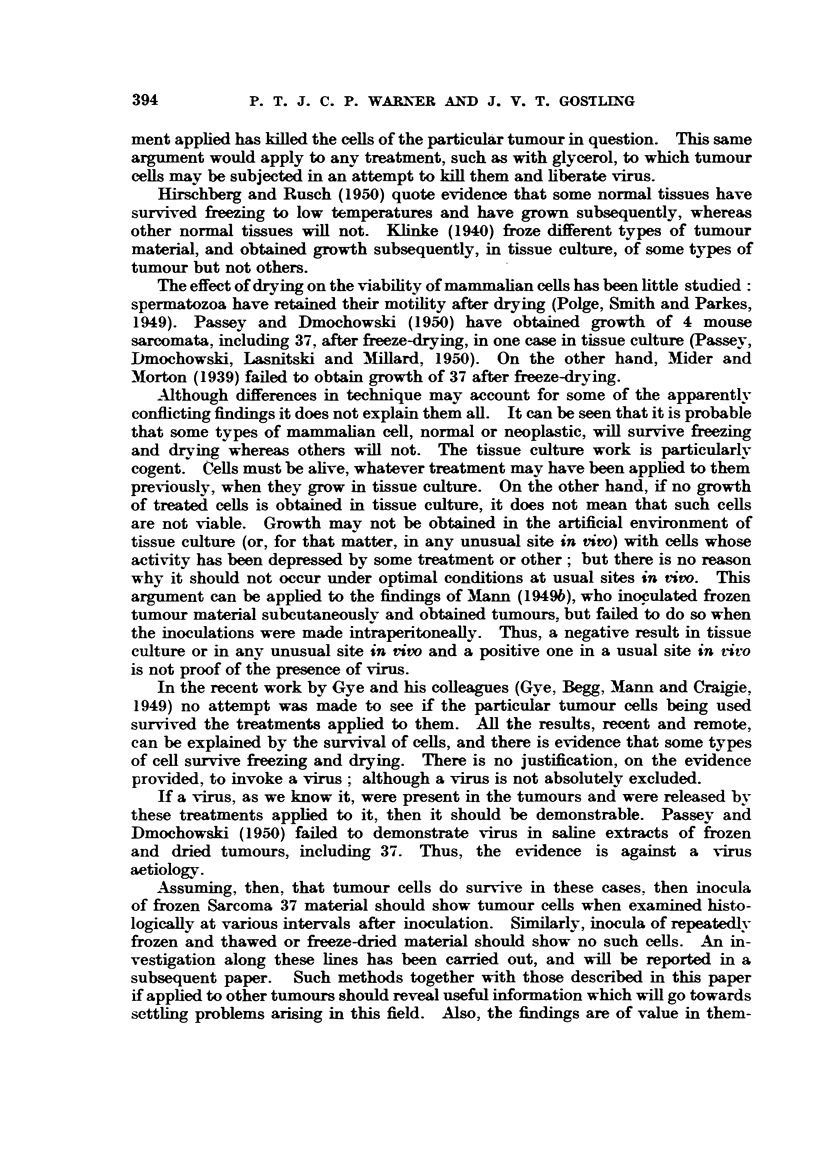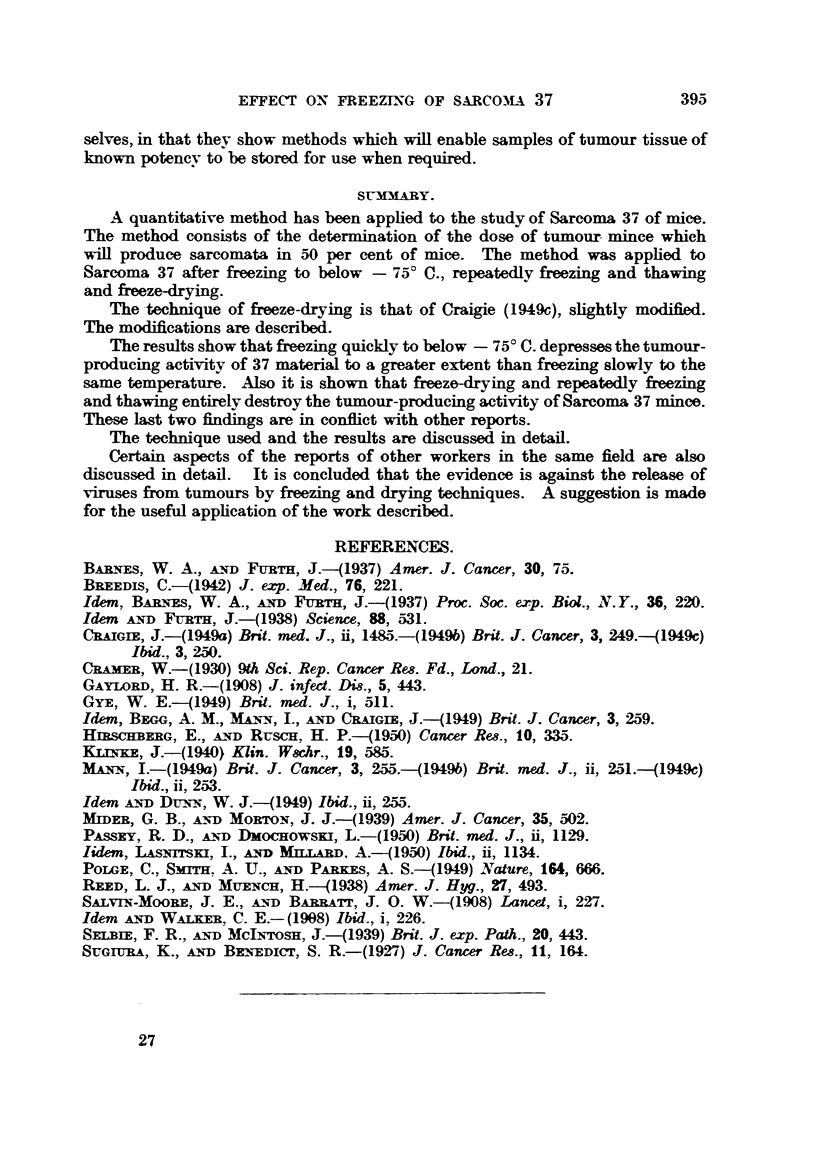# The Effect of Freezing and Freeze-drying on the Transplantation of Sarcoma 37

**DOI:** 10.1038/bjc.1950.37

**Published:** 1950-12

**Authors:** P. T. J. C. P. Warner, J. V. T. Gostling


### THE EFFECT OF FREEZING AND FREEZE-DRYING ON THE TRANSPLANTATION OF SARCOMA 37.

P. T. J. C. P. WARNER AND J. V. T. GOSTLING

*From the Bland-Sutton Institute of Pathology, The Middlesex Hospital, London, W.1.*

Received for publication November 17, 1950.

RECENTLY, Gye and his colleagues (Gye, 1949 ; Gye, Begg, Mann and Craigie, 1949 ; Mann, 1949*b*, 1949*c* ; Mann and Dunn, 1949) removed tumours from mice, exposed them to a temperature of — 79° C., and stored them at that temperature. They also dried them at room temperature and from the frozen state. Samples of tumour tissue after treatment in any one of these ways were inoculated into mice and tumours grew from a proportion of the inoculations. At the same time Mann (1949*a*) showed that mouse embryo tissue, which normally grew on inoculation into mice, would not do so after freezing at — 79° C. From this it was assumed that all cells, neoplastic or normal, were destroyed by the freezing treatments applied to them. As a result of this it was postulated that a virus-like agent was responsible for the tumour-producing activity of neoplastic tissue subjected to low temperature.

Somewhat similar claims were made as early as 1908 (Salvin-Moore and Walker, 1908 ; Salvin-Moore and Barratt, 1908 ; Gaylord, 1908), but the tumour tissue was subjected to low temperatures only ; drying was not used. However, Sugiura and Benedict (1927) noted that it was possible to transmit mammalian tumours with dried tissue. Subsequently, several reports appeared stating that tumour tissue exposed to low temperatures retained its tumour-producing activity (Cramer, 1930 ; Breedis, Barnes and Furth, 1937 ; Barnes and Furth, 1937 ; Breedis and Furth, 1938 ; Mider and Morton, 1939 ; Klinke, 1940 ; Breedis, 1942 ; Passey and Dmochowski, 1950). Some of these workers produced evidence that, among both normal and neoplastic cells, some types did and some did not survive exposure to low temperatures.

The contentions of Gye and his colleagues (Gye, 1949 ; Gye, Begg, Mann and Craigie, 1949 ; Mann, 1949*a*, 1949*b* ; Mann and Dunn, 1949) have been subjected to criticism on the grounds that they were based on insufficient evidence. One of the most recent and competent reviews of the subject is by Hirschberg and Rusch (1950). They conclude that the results do not prove that a virus was present in the tumours studied, but that they do not necessarily exclude it.

Passey and Dmochowski (1950), however, using various mouse tumours, including Sarcoma 37, were unable to demonstrate virus, by one of the usual methods, in tumour tissue which had been exposed to low temperatures. They also review previous work.

In view of the conflicting conclusions arrived at in this field, it was considered important to investigate the subject further. This paper deals with the application of quantitative methods, as suggested by Craigie (1949*a*), to Sarcoma 37 both before and after freeze-drying and exposure to temperatures below — 75° C.

MATERIALS AND METHODS.

*Sarcoma* 37.—This was obtained from the Courtauld Institute of Biochemistry, where it had been maintained in serial passage in albino mice.

*Animals.*—Albino mice were obtained from a reputable dealer. They were used at the age of 4 to 8 weeks and were weighed in groups before use. The average weight of the mice used for each experiment ranged from 16 to 26 g. Each mouse was earmarked.

*Preparation of sarcoma mince.*

Mice, bearing on their backs subcutaneous tumours, were killed, dipped in lysol and pinned, face-down, on a board. The size of the tumours ranged from 3 × 2 cm. to 0·5 × 0·8 cm., most being about 2 × 1 cm. The skin was cut at some distance from the tumour and around approximately three-quarters of its perimeter. The edges of the cut skin were everted and seized at two or three places by artery forceps. An assistant held the forceps and pulled the skin upwards and outwards to reveal the tumour. Sterile instruments were taken, and the tumour or the greater part of it was dissected away from its surroundings and placed in a weighed sterile Petri dish. A number of tumours were collected in this way. The Petri dish, containing the tumours, was then weighed. About 10 g. of tumour tissue was found to be a convenient amount. This was usually obtained from between 5 to 10 mice which had been inoculated with one minim (0·06 ml.) of minced tumour tissue 10 to 14 days previously.

After weighing, the tumours were cut up as finely as possible with scissors. The material was then minced in a pressure mincer similar to that described by Craigie (1949*b*), plungers with grooves of depths 0·02 in. and 0·005 in. being used. The fine mince was then placed in a 10 ml. syringe, and some of it was cultured on a blood agar plate.

*Titration of tumour-producing activity of sarcoma mince.*

The tumour mince described above was diluted in tenfold serial steps in physiological saline. The undiluted material and the dilutions were then inoculated in doses of 1 minim (0·06 ml.) at 4 different sites on each mouse used. A dose of 1 minim was chosen, for the sake of convenience, as the 1 ml. syringes used for inoculation were graduated in minims. The number of mice used for each dilution varied from experiment to experiment. The usual number was either 3 or 5, more frequently the latter.

The mice were examined at least twice a week and the number of progressively-growing tumours were recorded. From the results obtained the dose of tumour mince which would have produced tumours in 50 per cent of mice was calculated by the method of Reed and Muench (1938). This is referred to as the TPD50 (TPD = tumour-producing dose) of the mince. For the sake of clarity and convenience the negative logarithm of the TPD50 is shown in the results and is called the " TPD50 index."

*Quick freezing of sarcoma mince.*

Quantities of 1 ml. of sarcoma mince were fed, by means of a syringe, fitted with a long needle, into ampoules (6 mm. bore stems, 15·5 mm. diameter bulbs, obtainable from W. E. Edwards & Co., London). Each ampoule in turn was attached by a short stout piece of rubber tubing to the spindle of a 1/10 h.p. electric motor (Fig. 1) so that it was suspended vertically. The ampoule was allowed to hang within a brass tube whose diameter was 1 inch. The electric motor was started so that the ampoule rotated rapidly about its own longitudinal axis, and the tumour tissue was flung in a thin even layer on to the sides of the bulb. While the ampoule was still spinning, a beaker of alcohol, brought to a temperature of below — 75° C. by the addition of carbon dioxide ice, was raised so that it surrounded the ampoule and its contents. In this way the

FIG. 1.—Diagram of shell-freezing apparatus for tumour mince.

tumour mince was frozen in a thin shell on the inside of the ampoule. After freezing, the ampoule and its contents were stored at a temperature of below — 75° C. in alcohol and carbon dioxide ice contained in a vacuum flask. Usually the tumour mince was kept at this temperature for 1 hour, and, unless it was to be freeze-dried, the contents thawed in running tap water at a temperature of about 20° C. It was then titrated as described above.

On those occasions when quick-freezing preceded freeze-drying by Method II (see below), then tumour mince was placed in test tubes (1 in. × 6 in.) fitted with a B. 29 ground glass socket (B.S.S.). The test tubes were spun about their longitudinal axes and frozen as described for the ampoules. The test tubes were fitted with rubber bungs, in the upper surface of which holes were made to fit the spindle of the electric motor.

*Slow freezing of sarcoma mince.*

On some occasions tumour mince was placed in ampoules or test tubes 1 in. × 6 in. fitted with a ground glass socket (B.S.S., B. 29); these were spun about their longitudinal axis, as described under " Quick Freezing," in alcohol to which chips of carbon dioxide ice were added one by one, thus bringing the temperature down slowly, until the tumour mince was frozen. The tube or ampoule was then transferred to a thermos flask containing alcohol at the same temperature as that from which it was taken, and the slow freezing process continued until the temperature reached below − 75° C. This method was always used when slow freezing preceded freeze-drying, ampoules being used for Method I and test tubes for Method II (see below under the heading " Freeze-drying "). Because this method of slow-freezing was somewhat difficult in practice, on other occasions the mince was placed in a lump in ampoules or test tubes and the temperature brought down slowly as described above.

In both the methods of slow-freezing the temperature of the alcohol surrounding the test tubes or ampoules was taken at intervals. The temperature fell from between + 10° C. and + 20° C. to below − 75° C. over a period of time which ranged from 50 to 80 minutes in different experiments. The temperature fall is shown in Table I and Fig. 2. After the temperature had fallen below − 75° C. the tumour mince was allowed to remain in alcohol at the same temperature for a period of at least 1 hour. At the end of this time, unless it was to be freeze dried, it was thawed in running tap-water and titrated as described above.

TABLE I.—*Showing Temperatures at Five-minute Intervals During Five Slow Freezing Processes.*

| Time in minutes. | Temperature of alcohol surrounding ampoules, °C., in Experiment— | | | | |
|---|---|---|---|---|---|
| | 51 | 110 | 139 | 149 | 166 |
| 0 | 17 | 20 | 17 | 16 | 20 |
| 5 | 3 | 15 | 13 | 11 | 15 |
| 10 | −2 | 11 | 7 | 4 | 0 |
| 15 | −7 | 5 | −4 | −3 | −7 |
| 20 | −10 | 0 | −10 | −10 | −18 |
| 25 | −15 | −5 | −24 | −17 | −23 |
| 30 | −20 | −10 | −34 | −26 | −28 |
| 35 | −25 | −15 | −40 | −32 | −42 |
| 40 | −30 | −20 | −48 | −40 | −51 |
| 45 | −35 | −29 | −55 | −55 | −61 |
| 50 | −40 | −37 | −65 | −65 | −72 |
| 55 | −48 | −46 | −76 | −74 | .. |
| 60 | −50 | −54 | .. | .. | .. |
| 65 | −60 | −64 | .. | .. | .. |
| 70 | −66 | −73 | .. | .. | .. |
| 75 | −71 | −77 | .. | .. | .. |
| 80 | −77 | .. | .. | .. | .. |

This shows the temperature fall in 5 out of the 6 experiments in which slow freezing was carried out.

FIG. 2.—Graph of temperature fall in five slow-freezing experiments, showing temperatures at five-minute intervals.

| | | |
|---|---|---|
| Experiment | 51 | ——————— |
| „ | 110 | - - - - - - - - - |
| „ | 139 | —·—·—·— |
| „ | 149 | — — — — — — — |
| „ | 166 | —··—··—··— |

*Freeze-drying of sarcoma tissue.*

Freeze drying of sarcoma tissue was done by two methods. Apart from the device of freezing tumour mince in a shell by rapid spinning, described above, the method and apparatus used was fundamentally the same as that described by Craigie (1949c). In the first method used here a modification of the apparatus was made (Fig. 3), and in the second a modification of the method. The apparatus consisted of glass tubing and flasks whose bore was 1 in. or greater throughout. The different pieces were attached to one another by interchangeable ground-glass joints (B.S.S., B. 29). The apparatus was exhausted by a two-stage rotary pump (Model 2S 50, W. Edwards & Co.). The water-vapour was trapped by two condensers immersed in alcohol and carbon dioxide ice at a temperature of below — 75° C. The pressure within the apparatus was measured by a Pirani gauge (Model 5–2, W. Edwards & Co.) attached to the apparatus near the pump. Craigie's (1949c) vapour flow indicator was not used. Diagrams of the apparatus are shown in Fig. 3 and 4.

*Method I.*—In this method, Flask No. 3 (Craigie, 1949c, Fig. 1) was replaced by a flask of the same dimensions whose neck was fitted with 8 small side arms, bore 6 mm., instead of a single large bore side arm (Fig. 3). These side arms were fitted with pieces of stout rubber tubing which, when the apparatus was not in use, were stoppered with pieces of glass rod of a suitable diameter. Immediately preceding use the apparatus was evacuated until the Pirani gauge showed a reading of between 0·005 and 0 mm. Hg. When the ampoules of tumour mince, frozen in a shell and kept at below − 75° C., were ready, the rubber tubing attached to the side-arms was clipped with Spencer Wells forceps

FIG. 3.—Diagram of all-glass freeze-drying apparatus for use with ampoules.

and the glass rod stoppers removed. The ampoules were quickly attached to the rubber tubing and the Spencer Wells forceps removed. At this point the pressure within the apparatus rose rapidly, but fell again to its previous level within 5 minutes, at which it remained throughout the drying period. Pumping was continued for at least 3 hours, after which the ampoules were removed, sealed and stored overnight at room temperature. The following day the ampoules were opened and 1 ml. of distilled water was added. This reconstituted tumour mince was then titrated as described above.

*Method II.*—In this second method, tumour material in quantities of 3 ml. or less was placed in 6 in. × 1 in. test tubes fitted with B. 29 (B.S.S.) ground glass sockets. The tubes were spun about their vertical axis and the tumour material frozen in a thin shell as described above. Sometimes the freezing was carried out slowly and sometimes quickly according to the experiment. The tubes were then attached to the freeze-drying apparatus in its original unmodified form (Craigie, 1949c). The pump was allowed to run for at least 3 hours. During this time the tube containing the tumour mince was kept in an alcohol bath maintained at — 30° C. or below. No stirring mechanism was used; convection currents were relied upon to maintain an even temperature throughout the alcohol bath. At the end of the drying period the tube containing the dried tumour material was removed, lightly stoppered with cotton wool and placed in a desiccator over phosphorus pentoxide, where it was allowed to remain overnight. The following day a volume of distilled water was added equal to the original volume of material dried. The reconstituted material was then titrated.

FIG. 4.—Attachment to freeze-drying apparatus for use with test-tubes.

*Repeated freezing and thawing of sarcoma mince.*

Quantities of mince, each 1 ml., were placed in ampoules, which were quickly frozen while spinning about their vertical axis as already described. The ampoules were placed in alcohol at a temperature of below — 75° C. for a period of 5 minutes, and then placed in running tap water at about 20° C. until the contents were thawed. This process was repeated until a total of 6 freezings and thawings had been completed. The resulting material was then titrated.

### RESULTS.

Table II shows the results of slowly and quickly freezing sarcoma mince. This table shows in the first place that 37 sarcoma mince that had received no treatment whatsoever produced 100 tumours from 100 inoculations of 1

TABLE II.—*A Comparison of the Tumour-producing Activity of Untreated, Quickly Frozen and Slowly Frozen Minced Sarcoma* 37.

| Experiment. | Material. | Dilution. $10^{0}$ | $10^{-1}$ | $10^{-2}$ | $10^{-3}$ | $10^{-4}$ | TPD50 index. | Result of culture of mince on blood agar. |
|---|---|---|---|---|---|---|---|---|
| 41 | Untreated | 20/20 | 10/12 | 5/12 | 8/12 | .. | 3·6 | Micrococci ++ |
| | Quickly frozen and stored 1 day | 6/6 | 1/12 | 0/12 | 0/12 | .. | 1·8 | |
| | Quickly frozen and stored 16 days | 9/10 | .. | .. | .. | .. | — | |
| 51 | Untreated | 20/20 | 12/12 | 5/12 | 1/8 | .. | 3·1 | Coliform bacilli +++ |
| | Slowly frozen and stored 2 hours | 6/6 | 6/12 | 0/12 | 0/8 | .. | 2·2 | |
| | Slowly frozen and stored 10 days | 6/6 | 7/12 | 1/12 | 2/12 | .. | 2·5 | |
| 110 | Untreated | 20/20 | 17/20 | 7/20 | 1/20 | 0/20 | 3·0 | Sterile |
| | Quickly frozen | 10/10 | 17/20 | 6/20 | 1/20 | 1/20 | 2·9 | |
| | Slowly frozen | 20/20 | 20/20 | 16/20 | 8/20 | 3/20 | 4·0 | |
| 139 | Untreated | 20/20 | 16/20 | 4/20 | 1/16 | 0/16 | 2·7 | β-haemolytic streptococci ++ |
| | Quickly frozen | 15/15 | 10/20 | 0/20 | 0/20 | 1/20 | 2·2 | |
| | Slowly frozen | 20/20 | 10/12 | 1/20 | .. | .. | 2·6 | |
| 149 | Untreated | 20/20 | 20/20 | 2/20 | 5/20 | 2/20 | 2·9 | β-haemolytic streptococci ++ |
| | Quickly frozen | 20/20 | 2/8 | 0/12 | 0/20 | 0/20 | 1·9 | |
| | Slowly frozen | 20/20 | 20/20 | 4/20 | 0/4 | 0/8 | 2·8 | |
| 166 | Untreated | .. | 20/20 | 10/20 | 6/20 | 2/20 | 3·6 | β-haemolytic streptococci +++ |
| | Quickly frozen | 20/20 | 8/20 | 1/20 | 2/20 | 0/20 | 2·2 | |
| | Slowly frozen | 20/20 | 12/20 | 2/20 | 0/20 | 0/20 | 2·5 | |
| Totals each process from all experiments. | Untreated | 100/100 | 95/104 | 33/104 | 22/96 | 4/76 | 3·1 | |
| | Quickly frozen | 80/81 | 38/80 | 7/84 | 3/92 | 2/80 | 2·3 | |
| | Slowly frozen | 92/92 | 75/96 | 24/104 | 10/64 | 3/48 | 2·8 | |

1. All the materials used in any one experiment were prepared from the same mince.

2. Inoculum of undiluted or diluted material was 0·06 ml. in every case.

3. Denominators show number of inoculations made.

4. Numerators show number of sarcomata resulting.

5. TPD50 index = $x$ where the dose of mince which would be expected to cause sarcomata in 50 per cent of the mice is $10^{-x}$ ml.

6. .. = Not examined.

minim (0·06 ml.). With other unreported experiments the total number of 0·06 ml. doses of undiluted untreated mince was 612. From these 608 sarcomata resulted. Of the 4 mice in which no growth appeared, 2 were killed on the 7th and 2 on the 13th day because of intercurrent illness. All the tumours arose within 14 days as judged by the appearance of a progressively enlarging lump. The results of freezing Sarcoma 37 mince shown in Table II demonstrate clearly that, whether the mince had been frozen rapidly or slowly, its tumour-producing activity was not entirely destroyed. In fact undiluted mince treated in either way produced tumours in 172 cases out of 173—the one failure occurring in mince which had been frozen quickly and stored for 16 days (Table II, Experiment 41). The results of titrations show that in 3 cases (Experiments 51, 139, 149) out of 5 the slowly frozen mince was slightly less active than the control untreated material. In one case (Experiment 110) it was more active, and in the other (Experiment 116) it was considerably less active than the control material. In every experiment, however, the quickly frozen mince was less active than both the slowly frozen material (Experiments 110, 139, 149, 166) and the untreated control material (Experiments 41, 110, 139, 149, 166). The total figures for all experiments show that the TPD50 index of untreated material was 3·1, of quickly frozen material was 2·3, and the slowly frozen material 2·8. On two occasions (Experiments 41, 51) frozen mince was stored. In Experiment 41 the mince was frozen quickly and was stored for 16 days. No titration was performed on this material, but 9 tumours arose from 10 inoculations. In Experiment 51 the material was slowly frozen and stored for 10 days—the TPD50 index of this material was slightly greater than that of the same material which had only been stored for 2 hours.

All tumours which arose after freezing, slow or quick, appeared within 14 days, but did so in a slightly longer time than those arising from the corresponding untreated material.

Table II also shows the results of sterility tests. Only on one occasion was the mince found to be sterile (Experiment 110). In the first two experiments (Experiments 41, 51) the tests revealed contamination with micrococci in one and a coliform organism in the other. In the last three experiments (Experiments 139, 149, 166) the mince was contaminated with a β-haemolytic streptococcus. Although the minces were contaminated on each occasion all mice inoculated appeared to be perfectly fit and healthy, and the death rate among them was no greater than that occurring in the stock mice from which they were taken. Pathogenicity tests on mice were performed with the β-haemolytic streptococcus with negative results. Attempts to group the streptococcus have failed. Many attempts by various methods, including the use of antibiotics, have been made to free the mince from bacterial contamination without success.

Table III shows the result of inoculating tumour material which had been repeatedly (6 times) frozen and thawed. Four experiments were performed in this group. A total of 54 inoculations of frozen and thawed material was made. On no occasion did a progressively growing tumour arise. All mice were observed for a period of 35 days, and some of them were examined for longer periods up to 70 days. In the first two experiments (Experiments 8, 31) every single mouse inoculated with untreated control material developed tumours within 14 days. In the last two (Experiments 125, 184) no control experiments were performed. This was thought to be of no great importance, since past experience had shown that undiluted untreated material produced tumours in at least 99·3 per cent of cases (608 tumours from 612 inoculations).

TABLE III.—*Showing the Effect of Freezing and Thawing Six Times on the Tumour-producing Activity of Minced Sarcoma* 37.

| Experiment. | Tests. | | | | Controls. | | Result of culture of untreated mince on blood agar |
|---|---|---|---|---|---|---|---|
| | Number of mice. | Number of inocula. | Number of tumours. | Number of days observed. | Number of inocula. | Number of tumours. | |
| 8 | 5 | 5 | 0 | 36 | 50 | 50 | Coliform bacilli + + + |
| 31 | 3 | 3 | 0 | 36 | 20 | 20 | Sterile |
| 125 | 30 | 30 | 0 | 35–52 | .. | .. | ,, |
| 184 | 8 | 16 | 0 | 38–70 | .. | .. | Micrococci + |
| Totals | 46 | 54 | 0 | .. | 70 | 70 | |

Table IV shows the results of freeze-drying experiments. Of a total of 113 inoculations of freeze-dried material, not a single progressively growing tumour arose. In the first 5 experiments the material was frozen rapidly before drying, and in the last 5 it was frozen slowly. In Experiments 110*a*, 110*b* the material used was derived from the same tumour mince. However, slow freezing before freeze-drying did not affect the results. Except for one experiment (Experiment 149*a*), in which the mice died from an accident, all mice were examined for more than 36 days, mostly for longer periods up to 107 days (Experiment 139*a*). Out of the 10 experiments, all but 1 (Experiment 185) were performed in parallel with control observations made on untreated material from the same source. The results of sterility tests of the experiments summarized in Tables III and IV are shown in the appropriate place. On 4 occasions (Table III, Experiments 31, 125; Table IV, Experiments 110*a*, 110*b*) a sterile mince was obtained. On the other occasions the minces were contaminated to some degree or other.

In all the experiments summarized in Tables III and IV, although no progressively growing tumours were obtained, the inoculated material could be felt as a subcutaneous nodule during the first few days. These nodules gradually disappeared. In experiments not reported here, histological examination of such inocula of freeze-dried mince up to the 7th day revealed no evidence of tumour growth.

### DISCUSSION.

The results of the work reported here show, firstly, that freezing of Sarcoma 37 tissue does not deprive it of its tumour-producing activity, and that this property is less affected by slow freezing than by quick freezing (Table II). This finding is in agreement with those of Breedis, Barnes and Furth (1937) and Breedis and Furth (1938) who worked with leukaemic cells. Secondly, the results reported here show that repeated freezing and thawing on 6 occasions or drying from the frozen state deprived Sarcoma 37 tissue of all its tumour-producing activity.

One criticism that can be made of this work is that on most occasions the tumour minces investigated here were contaminated, to a greater or less degree,

TABLE IV.—*Showing the Effect of Freeze-drying on the Tumour-producing Activity of Minced Sarcoma 37.*

| Experiment. | Tests. Method of freezing. | Method of drying. | Number of mice. | Number of inocula. | Number of tumours. | Number of days observed. | Controls. Number of inocula. | Number of tumours. | Result of culture of untreated mince on blood agar. |
|---|---|---|---|---|---|---|---|---|---|
| 8a | Quick | I | 4 | 4 | 0 | 36 } | 50 | 50 | Coliform bacilli +++ |
| 8b | ,, | I | 3 | 3 | 0 | 43 } | | | |
| 41 | ,, | I | 5 | 10 | 0 | 56 | 20 | 20 | Micrococci ++ |
| 58 | ,, | I | 5 | 10 | 0 | 59 | 20 | 19 | Micrococci + |
| 110a | ,, | II | 5 | 10 | 0 | 39 } | 20 | 20 | Sterile |
| 110b | Slow | II | 5 | 10 | 0 | 39 } | | | |
| 139a | ,, | II | 4 | 16 | 0 | 107 | 20 | 20 | β-haemolytic streptococci ++ |
| 149a | ,, | II | 5 | 20 | 0 | 10 | 20 | 20 | β-haemolytic streptococci ++ |
| 166a | ,, | II | 6 | 12 | 0 | 75–82 | 20 | 20 | β-haemolytic streptococci +++ |
| 185 | ,, | II | 9 | 18 | 0 | 70 | .. | .. | Scanty micrococci |
| Totals | .. | .. | 51 | 113 | 0 | .. | 170 | 169 | |

TABLE V.—*Showing the Effect of Storage at* — 79° *C. on the Transplantation of Two Mouse Carcinomata:* (*Compiled from the Results of Mann,* 1949b, *Table I.*)

Tumour material was minced and then stored at – 79° C. for various periods of time. The stored material was then inoculated into mice and the number of tumours resulting were recorded.

| | Tumour "63" Inoculations. | Tumours. | Per cent tumours/inoculations. | Sporadic tumour. Inoculations. | Tumours. | Per cent tumours/inoculations. | Both tumours. Inoculations. | Tumours. | Per cent tumours/inoculations. |
|---|---|---|---|---|---|---|---|---|---|
| Storage for 48 hrs. and less | 84 | 37 | 44 | 38 | 35 | 92 | 122 | 72 | 59 |
| Storage for more than 48 hrs. | 24 | 12 | 50 | 79 | 70 | 89 | 103 | 82 | 80 |
| Totals | 108 | 49 | 45 | 117 | 105 | 90 | 225 | 154 | 69 |

with bacteria (Tables II, III, IV). However, in most of the experiments on freezing and repeated freezing and thawing such tumour material was kept for only a short period of time before reinoculation, and during that time it was maintained at a temperature of below $-75°$ C. The mice into which the contaminated tumour material was inoculated showed no signs of constitutional disturbance, and in most cases adequate control inoculations were made of material from the same source as that used in each experiment. In these cases it was felt that bacterial contamination was of no significance. In the case of the freeze-drying experiments (Table IV) material was kept in the dried state for 24 hours before reconstitution and reinoculation. Under these conditions it is just conceivable that the products of bacterial metabolism might have an effect on the tumour-producing activity of the freeze-dried material. However, this is thought to be unlikely, especially in those experiments where the tumour material was subjected to drying, primary and secondary throughout the 24 hours by Method II of freeze-drying. Every attempt was made to eliminate bacterial infection, and it is obviously desirable to do so. However, provided that the degree of infection is noted, it does not necessarily detract from the value of the observations.

The quantitative method described in this paper was chosen rather than one based on the time of first appearance of tumours, for the reason that the time of first appearance of a tumour was difficult to determine. The original inoculum of tumour material could, in nearly every case, be felt immediately after inoculation. Every inoculum showed a slight initial enlargement, even if it had been made with material which had been inactivated by repeated freezing and thawing or freeze-drying. This enlargement was shown by histological methods to be due to an inflammatory reaction which on occasions was not due to bacteria, because it occurred with samples of material which were bacteriologically sterile. Thus, it was impossible to know, by palpation, where inflammatory reaction ended and sarcoma growth began. Initially, a quantitative method based on the rate of growth of tumours as judged by caliper measurements was tried. This was found to be subject to immense variation even when a large number of observations were performed. Thus the only practicable method available was one such as is described here. It has no pretensions of great accuracy, because it is technically difficult to obtain a dispersion of tumour mince to the same degree as can be obtained with viruses. It is claimed, however, that the method described here when used on a sufficiently large scale is more accurate than those used heretofore.

It is to be noted that tumour mince was diluted immediately before titration. All treatments used in this work were applied to undiluted mince, and thus the variable effect of diluents has been entirely eliminated. Also the technique of freezing tumour mince in a thin shell in standard-sized ampoules or test tubes has ensured that exposure to cold or drying processes has been as nearly strictly comparable as possible. Only in the case of slow freezing was tumour tissue exposed to cold in a lump, but here the only purpose was to ensure that the temperature of the tumour mince fell slowly. The fact that the temperature of some parts of the mince may have fallen at a slower rate than others was not of great consequence, because the purpose of the experiment was to distinguish between quickly frozen material and slowly frozen material, and not to draw conclusions, at this point, about the effect of the rate of fall of temperature.

The effect of cold on Sarcoma 37 has not been widely studied. Gye, Begg, Mann and Craigie (1949) used this tumour in one experiment, where it was stored without diluent at — 78° C. for a period of 201 days. Five inoculations of this frozen and stored material produced 5 tumours in 7 days. This is not at variance with the findings reported here (Table II). Passey and Dmochowski (1950) report similar results.

Cramer (1930) was successful in obtaining tumours from grafts of material which had been repeatedly frozen and thawed either 4 or 8 times. This differs from the findings here (Table III); however, Cramer's technique differed from that used here. In one experiment Cramer exposed his material to a temperature of — 20° C. In the other he used a temperature of at least — 80° C., but exposed his tumour material to it in a mass contained in a test tube. Under this condition the destructive effect of repeated freezing and thawing may have been diminished. The difference in technique may account for the conflicting results. In addition it is possible that the properties of Sarcoma 37 vary from time to time, and that the strain of mice used may affect the outcome of experiments with this tumour.

Mider and Morton (1939) also using 37 stated that they were able to obtain tumours in 10 per cent of cases after freezing and thawing the tumour tissue *en bloc* 4 times. The number of mice used in their experiments is not stated. Selbie and McIntosh (1939) were unable to obtain growth from Sarcoma 37 tissue which had been repeatedly frozen and thawed.

Mider and Morton (1939) dried Sarcoma 37 from the frozen state and failed to obtain tumours from the dried material. However, Passey and Dmochowski (1950) were able to obtain growth from dried material of the same tumour desiccated in 5·3 per cent glucose solution. The use of the glucose solution may account for the fact that these findings are at variance with those reported here; no diluent was used during desiccation in the work reported in the present paper.

The first method (Method I) of freeze-drying used here was less efficient than that used by Gye, Begg, Mann and Craigie (1949), but the second method was more so. Firstly, because the tumour mince was spread thinly and evenly over the wall of the large test-tubes, and secondly, because secondary drying over phosphorus pentoxide was carried out. Despite this added efficiency no tumours were obtained.

It should be noted that none of the tumour mince used here was stored in the frozen state for any length of time before freeze-drying; this is of importance, because Gye, Begg, Mann and Craigie (1949) state that they obtained a greater number of tumours with material which had been stored at — 78° C. for some time before freeze-drying. There is no evidence provided for this statement, but Mann (1949*b*) says that tumour material which had been stored at — 78° C. for longer than 48 hours produced more tumours than that which had been stored for a shorter period. In view of the implications of this statement it is thought worth while to review the details of her results. If Mann's tabulated results (Mann, 1949*b*, Table I) are examined, it will be noted that she used two different tumours, "63" and "Sporadic." Her results, excluding those experiments (M60A, M65 and M72) in which technical errors occurred and which she herself excluded in her analysis, are re-tabulated in Table V in such a way that each tumour is considered separately. It will be seen that the total incidence of tumours derived from " 63 " frozen material, stored for whatever length of time, is 45 per cent (49 tumours from 108 inoculations), and for those observations made on tumour material stored for less than 48 hours it is 44 per cent (37 tumours from 84 inoculations). For those observations made on tumour material stored for more than 48 hours the incidence of tumours is 50 per cent (12 tumours from 24 inoculations). The difference between the percentage incidences before and after 48 hours' storage (44 per cent and 50 per cent respectively) is not statistically significant (Standard Error of difference = 11·5 per cent). Similarly, with the " Sporadic " tumour: the percentage incidence for all frozen Sporadic material is 90 per cent (105 tumours from 117 inoculations), that for material stored for less than 48 hours is 92 per cent (35 tumours from 38 inoculations), and that stored for more than 48 hours is 89 per cent (70 tumours from 79 inoculations). Again and more obviously the difference between the incidences before and after 48 hours' storage is not significant (Standard Error of the difference = 5·6 per cent).

Further examination of Mann's (1949*b*) table shows that in each case the incidence of tumours resulting from the inoculation of frozen and stored material is approximately the same for whatever period of time storage was carried out. In the case of tumour " 63 " this will be 45 $\pm$ 9·6 per cent, and for " Sporadic " it will be 90 $\pm$ 5·6 per cent. If account is taken of this and the fact that in the first 48 hours most of Mann's (1949*b*) observations (84 out of a total of 122) were made on tumour " 63," and after 48 hours most observations (79 out of 103) were made on the " Sporadic " tumour, then her conclusions are explained. Clearly, the conclusions are based on an initial bias in the form of an unequal weighting of material which vitiates subsequent statistical analysis.

It was considered important to deal with this matter is detail here for four reasons :

1. Because the statement arising from the results as presented by Mann (1949*b*) is misleading.
2. The results, as interpreted here, suggest that the storage of a particular tumour at − 79° C. for any period of time up to 182 days does not greatly affect the tumour-producing activity of the material.
3. The results, as interpreted here, suggest the possibility that freezing to low temperatures affects the tumour-producing activities of different tumours to different degrees.
4. From " 2 " it may follow that freezing to − 79° C. is a convenient method of storing standard samples of tumour material.

As a result of the above it was considered that there was no reason to believe that there was necessarily an advantage in storing tumour material below − 75° C. before desiccation. Therefore this practice was not adopted in the present investigation. However, the figures shown in Table I suggest that there would be an advantage in slowly freezing Sarcoma 37 material before freeze-drying. This is based on the assumption that the more active the starting material the more likely it would be to survive the freeze-drying process. However, this manoeuvre failed to produce material which was active after freeze-drying (Table IV).

Whether or not the induction of tumours by neoplastic tissue which has been frozen or dried implies causation by virus depends upon whether the treatment applied has killed the cells of the particular tumour in question. This same argument would apply to any treatment, such as with glycerol, to which tumour cells may be subjected in an attempt to kill them and liberate virus.

Hirschberg and Rusch (1950) quote evidence that some normal tissues have survived freezing to low temperatures and have grown subsequently, whereas other normal tissues will not. Klinke (1940) froze different types of tumour material, and obtained growth subsequently, in tissue culture, of some types of tumour but not others.

The effect of drying on the viability of mammalian cells has been little studied : spermatozoa have retained their motility after drying (Polge, Smith and Parkes, 1949). Passey and Dmochowski (1950) have obtained growth of 4 mouse sarcomata, including 37, after freeze-drying, in one case in tissue culture (Passey, Dmochowski, Lasnitski and Millard, 1950). On the other hand, Mider and Morton (1939) failed to obtain growth of 37 after freeze-drying.

Although differences in technique may account for some of the apparently conflicting findings it does not explain them all. It can be seen that it is probable that some types of mammalian cell, normal or neoplastic, will survive freezing and drying whereas others will not. The tissue culture work is particularly cogent. Cells must be alive, whatever treatment may have been applied to them previously, when they grow in tissue culture. On the other hand, if no growth of treated cells is obtained in tissue culture, it does not mean that such cells are not viable. Growth may not be obtained in the artificial environment of tissue culture (or, for that matter, in any unusual site *in vivo*) with cells whose activity has been depressed by some treatment or other ; but there is no reason why it should not occur under optimal conditions at usual sites *in vivo*. This argument can be applied to the findings of Mann (1949*b*), who inoculated frozen tumour material subcutaneously and obtained tumours, but failed to do so when the inoculations were made intraperitoneally. Thus, a negative result in tissue culture or in any unusual site *in vivo* and a positive one in a usual site *in vivo* is not proof of the presence of virus.

In the recent work by Gye and his colleagues (Gye, Begg, Mann and Craigie, 1949) no attempt was made to see if the particular tumour cells being used survived the treatments applied to them. All the results, recent and remote, can be explained by the survival of cells, and there is evidence that some types of cell survive freezing and drying. There is no justification, on the evidence provided, to invoke a virus ; although a virus is not absolutely excluded.

If a virus, as we know it, were present in the tumours and were released by these treatments applied to it, then it should be demonstrable. Passey and Dmochowski (1950) failed to demonstrate virus in saline extracts of frozen and dried tumours, including 37. Thus, the evidence is against a virus aetiology.

Assuming, then, that tumour cells do survive in these cases, then inocula of frozen Sarcoma 37 material should show tumour cells when examined histologically at various intervals after inoculation. Similarly, inocula of repeatedly frozen and thawed or freeze-dried material should show no such cells. An investigation along these lines has been carried out, and will be reported in a subsequent paper. Such methods together with those described in this paper if applied to other tumours should reveal useful information which will go towards settling problems arising in this field. Also, the findings are of value in themselves, in that they show methods which will enable samples of tumour tissue of known potency to be stored for use when required.

SUMMARY.

A quantitative method has been applied to the study of Sarcoma 37 of mice. The method consists of the determination of the dose of tumour mince which will produce sarcomata in 50 per cent of mice. The method was applied to Sarcoma 37 after freezing to below − 75° C., repeatedly freezing and thawing and freeze-drying.

The technique of freeze-drying is that of Craigie (1949c), slightly modified. The modifications are described.

The results show that freezing quickly to below − 75° C. depresses the tumour-producing activity of 37 material to a greater extent than freezing slowly to the same temperature. Also it is shown that freeze-drying and repeatedly freezing and thawing entirely destroy the tumour-producing activity of Sarcoma 37 mince. These last two findings are in conflict with other reports.

The technique used and the results are discussed in detail.

Certain aspects of the reports of other workers in the same field are also discussed in detail. It is concluded that the evidence is against the release of viruses from tumours by freezing and drying techniques. A suggestion is made for the useful application of the work described.

REFERENCES.

Barnes, W. A., and Furth, J.—(1937) *Amer. J. Cancer*, **30**, 75.

Breedis, C.—(1942) *J. exp. Med.*, **76**, 221.

*Idem*, Barnes, W. A., and Furth, J.—(1937) *Proc. Soc. exp. Biol., N.Y.*, **36**, 220.

*Idem* and Furth, J.—(1938) *Science*, **88**, 531.

Craigie, J.—(1949*a*) *Brit. med. J.*, ii, 1485.—(1949*b*) *Brit. J. Cancer*, **3**, 249.—(1949*c*) *Ibid.*, **3**, 250.

Cramer, W.—(1930) *9th Sci. Rep. Cancer Res. Fd., Lond.*, 21.

Gaylord, H. R.—(1908) *J. infect. Dis.*, **5**, 443.

Gye, W. E.—(1949) *Brit. med. J.*, i, 511.

*Idem*, Begg, A. M., Mann, I., and Craigie, J.—(1949) *Brit. J. Cancer*, **3**, 259.

Hirschberg, E., and Rusch, H. P.—(1950) *Cancer Res.*, **10**, 335.

Klinke, J.—(1940) *Klin. Wschr.*, **19**, 585.

Mann, I.—(1949*a*) *Brit. J. Cancer*, **3**, 255.—(1949*b*) *Brit. med. J.*, ii, 251.—(1949*c*) *Ibid.*, ii, 253.

*Idem* and Dunn, W. J.—(1949) *Ibid.*, ii, 255.

Mider, G. B., and Morton, J. J.—(1939) *Amer. J. Cancer*, **35**, 502.

Passey, R. D., and Dmochowski, L.—(1950) *Brit. med. J.*, ii, 1129.

*Iidem*, Lasnitski, I., and Millard, A.—(1950) *Ibid.*, ii, 1134.

Polge, C., Smith, A. U., and Parkes, A. S.—(1949) *Nature*, **164**, 666.

Reed, L. J., and Muench, H.—(1938) *Amer. J. Hyg.*, **27**, 493.

Salvin-Moore, J. E., and Barratt, J. O. W.—(1908) *Lancet*, i, 227.

*Idem* and Walker, C. E.—(1908) *Ibid.*, i, 226.

Selbie, F. R., and McIntosh, J.—(1939) *Brit. J. exp. Path.*, **20**, 443.

Sugiura, K., and Benedict, S. R.—(1927) *J. Cancer Res.*, **11**, 164.